# Supplementary material for: ADAR1 Suppresses Interferon Signaling in Gastric Cancer Cells by MicroRNA-302a-Mediated IRF9/STAT1 Regulation
Source: Int J Mol Sci. 2020 Aug 27;21(17):6195. doi: 10.3390/ijms21176195 (PMC7504523; doi:10.3390/ijms21176195)

# Supplementary Information

## Supplementary Figure Legends

**Supplementary fig. 1.** qRT-PCR (A) and immunoblot (B) analysis of ADAR1 expression in AGS after stable knockdown of ADAR1 in AGS cells using shRNA against 3' UTR of ADAR1 or control (shGFP).

**Supplementary fig. 2.** Quantitation of western blots shown in Figure 2C. Densitometry data obtained by ImageJ software are presented as relative value to control (untreated). ADAR1(A), STAT1(B), p-STAT1(C), STAT2(D), p-STAT2(E) and IRF9(F) are shown.

**Supplementary fig. 3.** Quantitation of western blots shown in Figure 3F. Densitometry data obtained by ImageJ software are presented as relative value to control (untreated). ADAR1(A), STAT1(B) and STAT1(C) are shown.

**Supplementary fig. 4** (A) Sequence alignment showing the miR-302a-5p seed sequence interacting with the 3'-UTR of STAT1 mRNA. (B) Luciferase assay result using wild-type STAT1 UTR reporter (WT) or mutant with the seed-binding site of miR-302a-5p (MUT), co-transfected with miR-302a-5p mimic or scrambled control.

**Supplementary fig. 5.** MAVS is edited by ADAR1 but its protein, mRNA level or localization are not affected by ADAR1 knockdown. (A) Sanger sequencing result of two MAVS editing sites (chr20:3850513, chr20:3850514) in genomic DNA, control cDNA and ADAR1 KD cDNA. Arrows indicate A-to-G editing sites. (B-C) qRT-PCR (B) and immunoblot (C) of MAVS upon ADAR1 knockdown. (D) Immunofluorescence image of MAVS upon ADAR1 knockdown. Mitotracker images are shown as green images.

**Supplementary fig. 6.** IFNAR2 is edited by ADAR1 but its protein, mRNA level or localization are not affected by ADAR1 knockdown. (A) Sanger sequencing result of IFNAR2 (chr21; 34636384) for genomic DNA, control cDNA and ADAR1 knocked-down cDNA. Arrows indicate A-to-G editing sites. Immunofluorescence image of IFNAR2 (green on top) upon ADAR1 knockdown. (B) Immunofluorescence image of IFNAR2 upon ADAR1 knockdown. No detectable change in the localization of IFNAR2 protein was observed in the absence or presence of IFN.

**Supplementary fig. 7.** ADAR1 does not affect two known mechanisms of STAT1 protein stabilization (A) Sequencing results of STAT1 cDNA sequence at phenylalanine 172 in AGS shGFP and AGS shADAR1 cell. (B) Sequencing results of STAT1 cDNA sequence at N92 coding region from AGS shGFP and AGS shADAR1 cell. The black peak indicated by arrow on left panel shows edited signal. (C) Immunoblot results of protein PKR, P-PKR, eIF-2α and STAT1α / STAT1β in AGS control and AGS ADAR1 KD cells.

**Supplementary fig. 8.** miR RT-PCR result showing detectable amount of miR-302a-3p only in AGS cell line among seven cancer cell lines examined.

**Supplementary Figure 1**

A

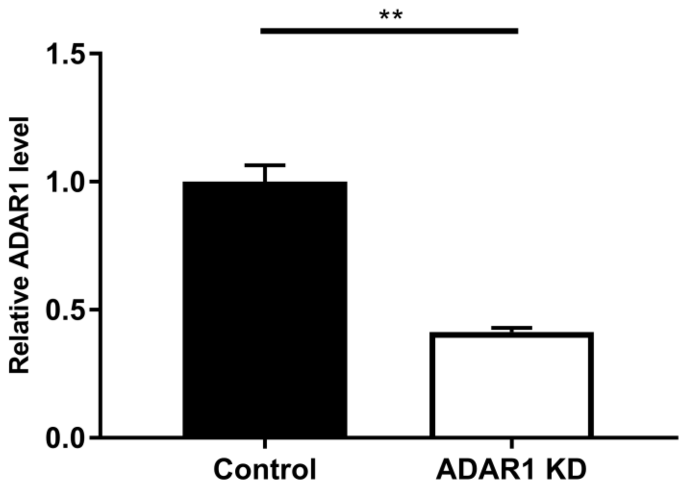


B

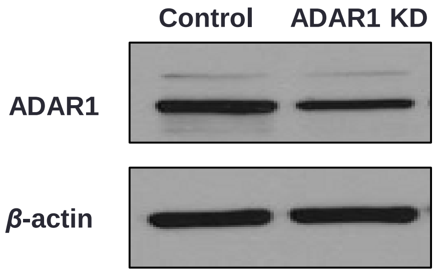

Supplementary Figure 2

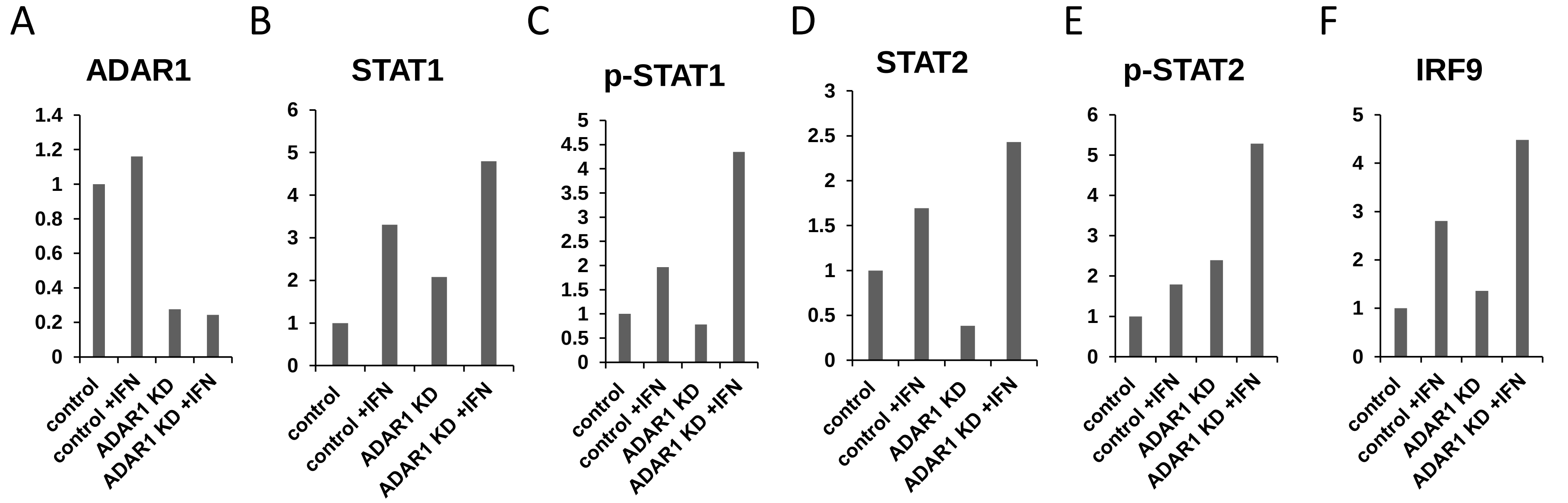

**Supplementary Figure 3**

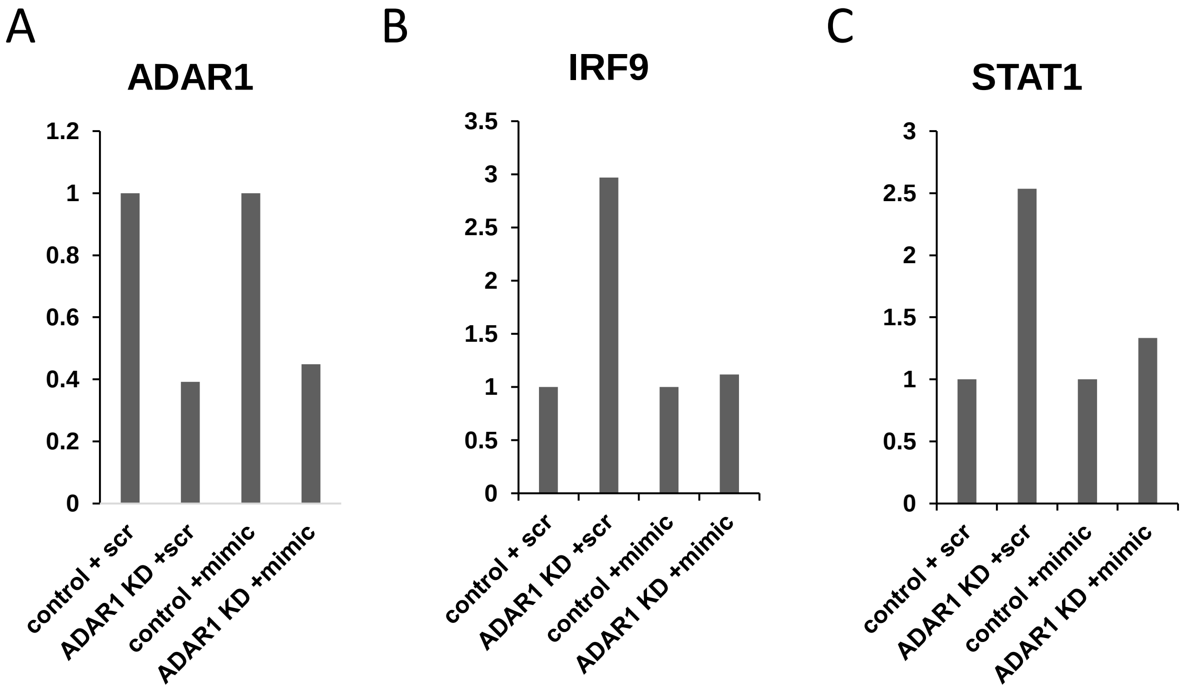

**Supplementary Figure 4**

A

**Position 3219-3225 of STAT1 3’ UTR** 5’ …CCAAGAGACUUUACU**UUUAAG**AA…

**I I I I I I**

**Hsa-miR-302a-5p** 3’ UCGUUCAUGUAGGUGC**AAAUUC**A

B

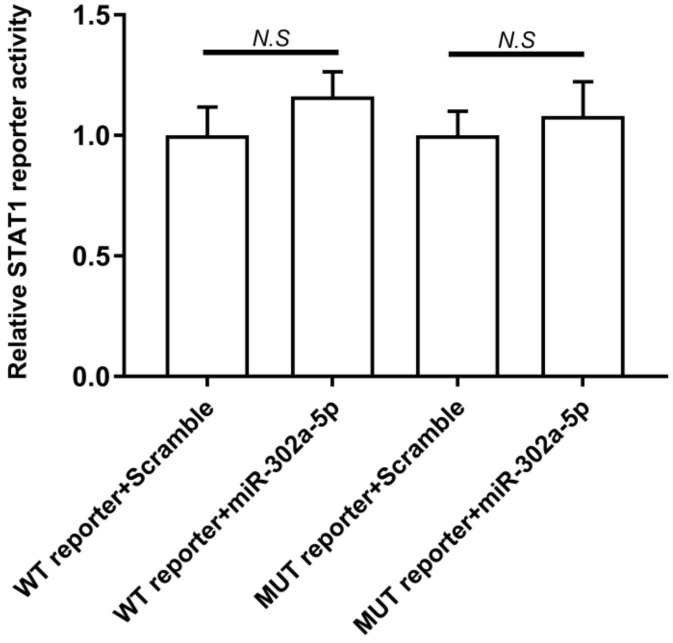

## Supplementary Figure 5

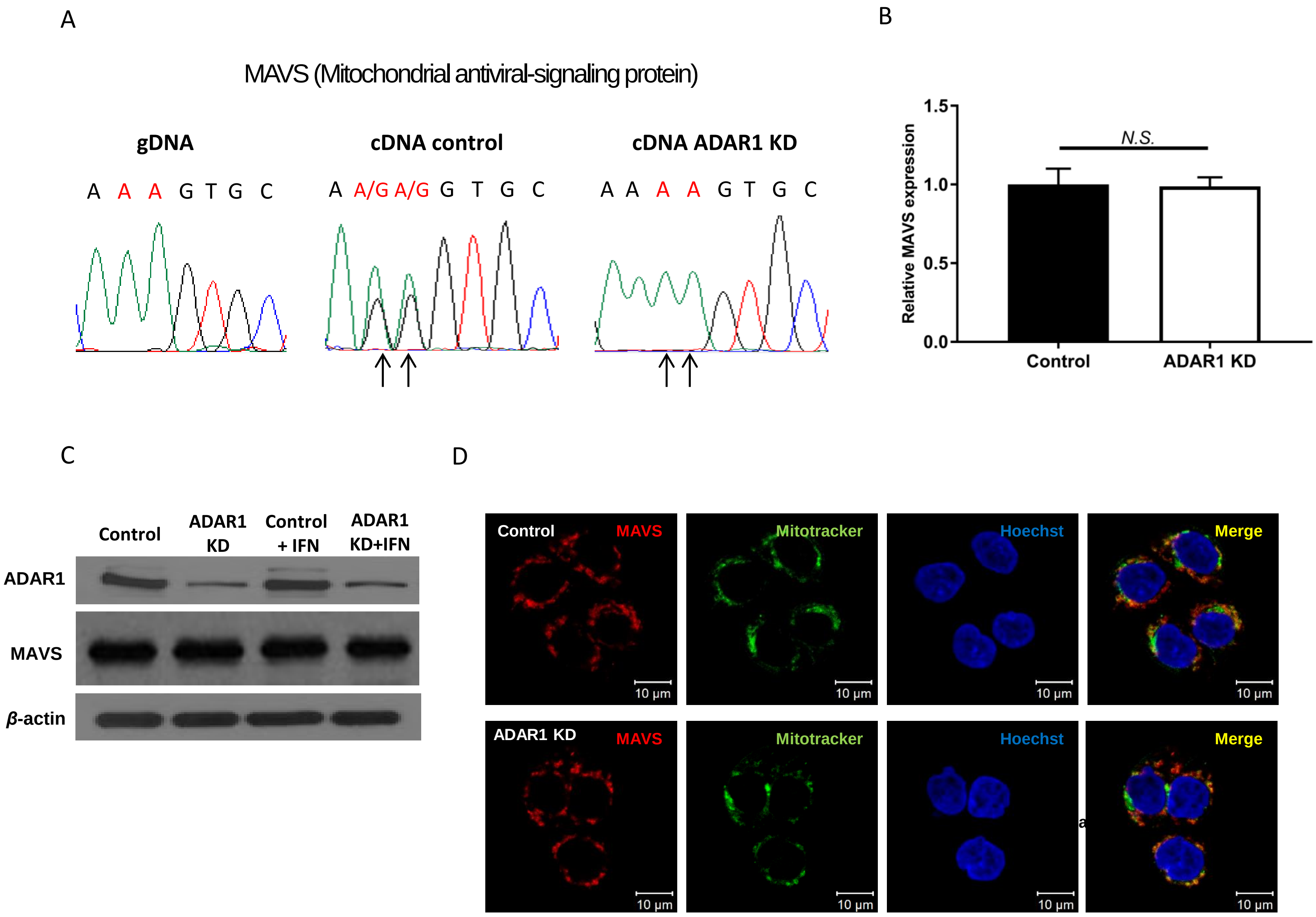

**Supplementary Figure 6**

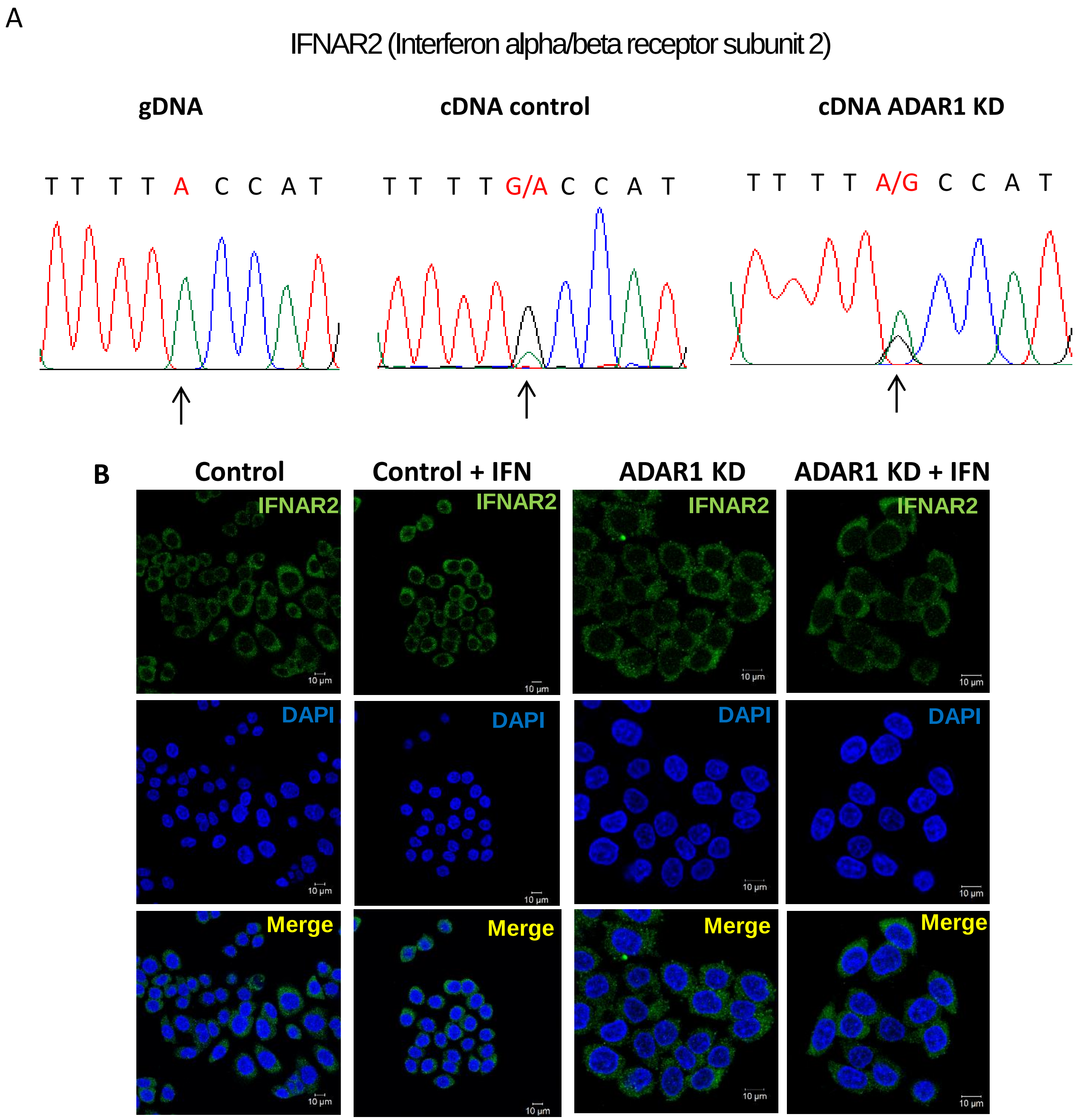

**Supplementary Figure 7**

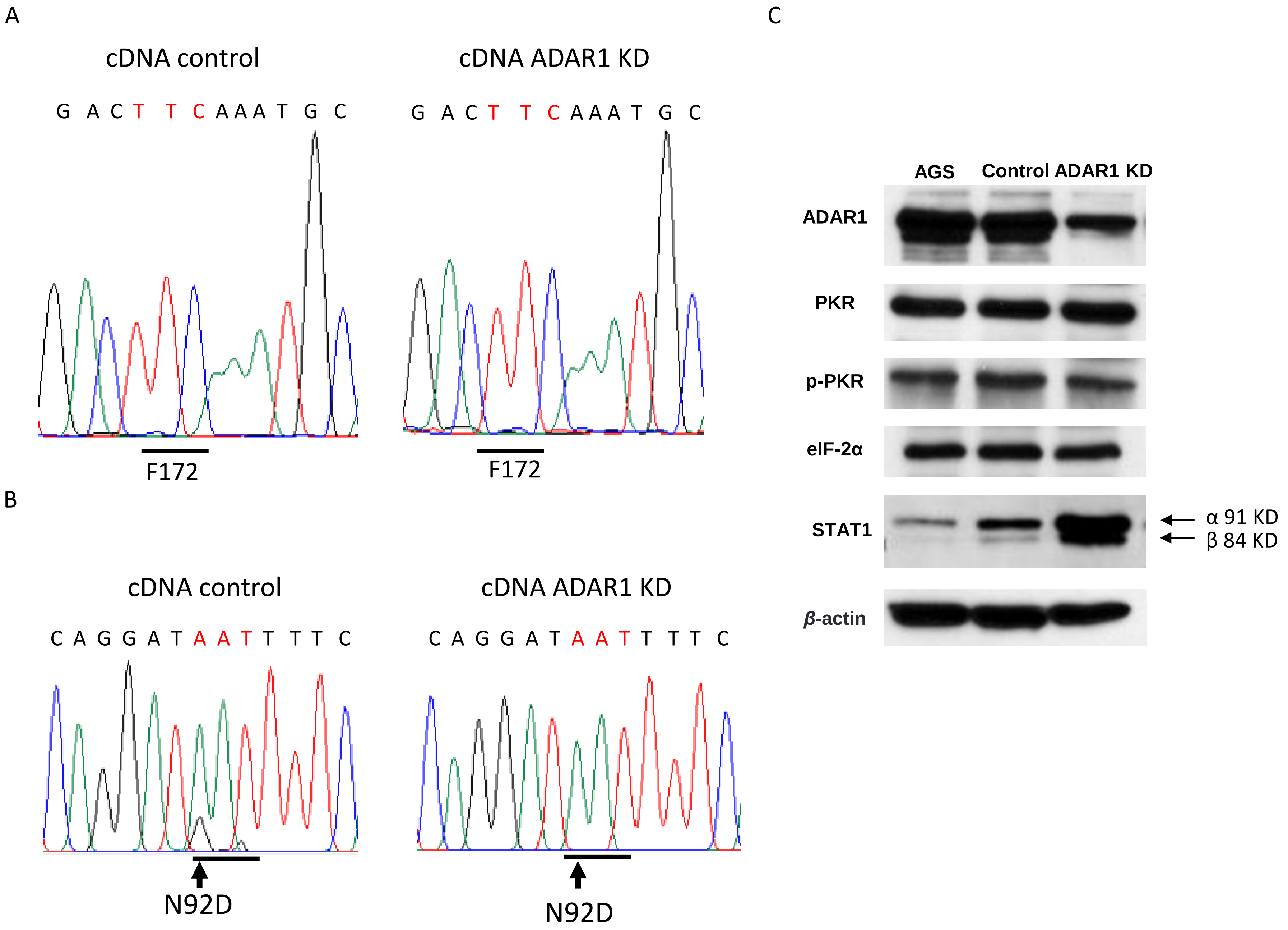

**Supplementary Figure 8**

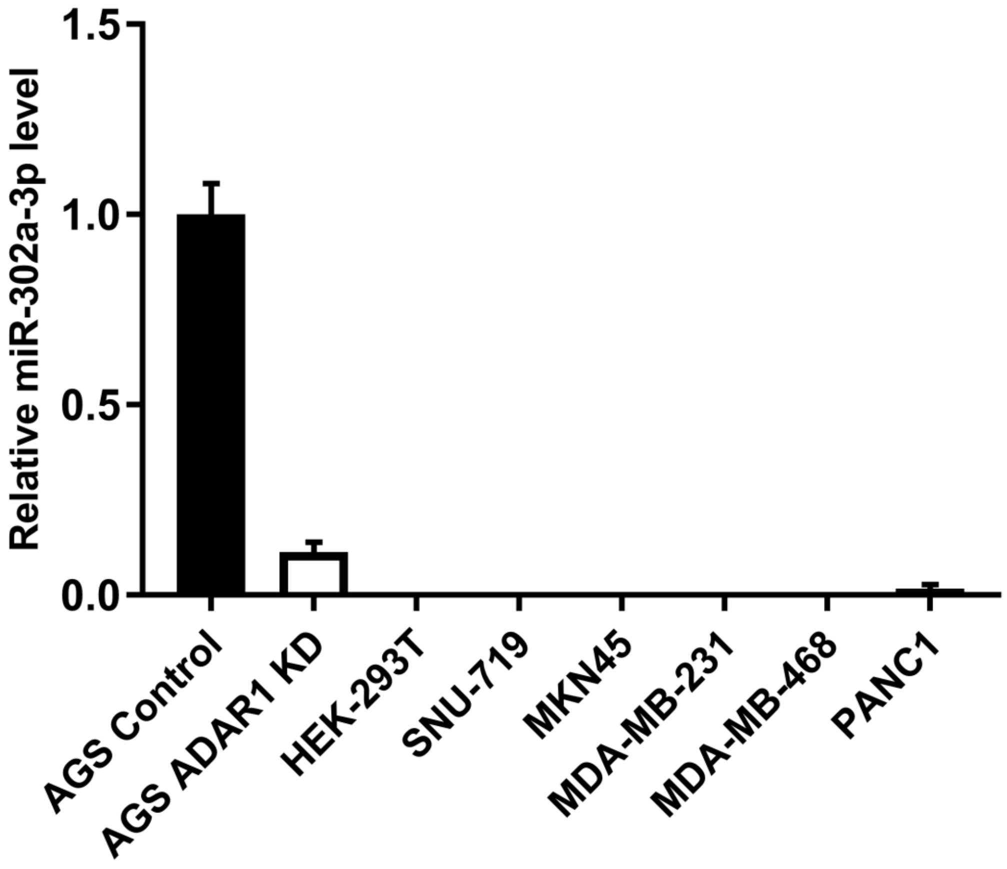

Supplement: Supplementary file 1 [file ijms-21-06195-s001.pdf]
